# From ecology to evolution: plasmid- and colicin-mediated persistence of antibiotic-resistant *Escherichia coli* in gulls

**DOI:** 10.1128/msystems.01663-25

**Published:** 2025-12-29

**Authors:** Michaela Ruzickova, Jana Palkovicova, Kristina Nesporova, Marketa Rysava, Rene Pariza, Simon Krejci, Ivan Literak, Monika Dolejska

**Affiliations:** 1 Department of Biology and Wildlife Diseases, University of Veterinary Sciences Brno 48358, Brno, Czech Republic; 2 Central European Institute of Technology, University of Veterinary Sciences Brno 48358, Brno, Czech Republic; 3 Department of Microbiology, Faculty of Medicine in Pilsen, Charles University 60569, Pilsen, Czech Republic; 4 Division of Clinical Microbiology and Immunology, Department of Laboratory Medicine, University Hospital Brno 48358, Brno, Czech Republic; 5 Department of Chemistry and Biochemistry, Faculty of AgriSciences, Mendel University in Brno 48269, Brno, Czech Republic; University of Technology Sydney, Sydney, NSW, Australia

**Keywords:** *Escherichia coli*, IncI1 plasmid, IncF plasmid, gulls, environment, antibiotic resistance, colicin

## Abstract

Antimicrobial resistance (AMR) in wildlife is an emerging concern within the One Health concept. Gulls, due to their synanthropic behavior and long-distance migration, are recognized as vectors and secondary reservoirs of resistant bacteria. These birds can facilitate the environmental spread of resistant strains across ecosystem boundaries. Understanding their role in shaping microbial communities is essential for assessing the broader ecological impact. This study investigates the persistence and competitive dynamics of cephalosporin-resistant *Escherichia coli* in Caspian gulls (*Larus cachinnans*) captured at their breeding colony at a water reservoir and subsequently monitored in captivity for three months, representing the longest *in vivo* experiment of its kind conducted on wild birds. We observed sustained colonization and long-term shedding of resistant *E. coli* throughout the entire study, marking the longest documented carriage of resistant bacteria in wild birds to date. Notably, rapid dissemination of various *E. coli* sequence types (STs) with CTX-M-1 was observed, with ST11138 rapidly outcompeting other strains, including the initially dominant ST11893. Genomic analyses revealed that ST11138 harboured F24:A-:B1 and IncI1/ST3/CTX-M-1 plasmids encoding colicins and corresponding immunity genes, likely conferring a competitive advantage. Our findings underscore the role of bacteriocin-mediated interactions in shaping microbial communities and highlight the importance of plasmid-encoded traits in the persistence of resistant strains in wildlife. Importantly, our findings underscore the ecological novelty of longitudinal *in vivo* tracking of AMR persistence in natural hosts and highlight the need to consider ecological and microbiome-level interactions when assessing the environmental dimension of AMR under the One Health concept.

**IMPORTANCE:**

Antimicrobial resistance (AMR) in wildlife is an emerging concern within the One Health framework, with gulls recognised as important vectors and secondary reservoirs of resistant bacteria. Due to their synanthropic behavior and long-distance migration, these birds can facilitate the spread of resistant strains across ecosystems. However, the role of wildlife in resistance dynamics remains underexplored, especially in long-term, natural settings. Our study is unique in its scope and duration, representing the longest *in vivo* experiment of its kind conducted on wild birds. By capturing these processes in live hosts under naturalistic conditions and across an extended period, our study provides rare and ecologically grounded insights into how AMR is maintained outside clinical or laboratory settings. Our findings show sustained colonisation and long-term shedding of resistant *E. coli*, with strain ST11138 outcompeting others. Genomic analyses reveal plasmid-encoded traits, highlighting the ecological and evolutionary mechanisms underlying resistance maintenance in wildlife.

## INTRODUCTION

Antimicrobial resistance (AMR) is a global concern with profound implications for human and veterinary medicine, agriculture, and environmental health (1). While the role of clinical and agricultural settings in driving AMR is well established, the environmental dissemination of resistant bacteria is increasingly recognised as a critical component (2, 3). In particular, the spread of AMR bacteria into natural ecosystems has been closely associated with synanthropic species, which thrive in human-altered environments and may acquire resistant bacterial strains through contact with anthropogenically impacted habitats (3, 4). Migratory birds, such as gulls (*Laridae*), are among the most effective biological vectors of AMR due to their long-distance movements and opportunistic foraging behavior. These birds frequently exploit sites such as landfills, sewage outflows, and agricultural landscapes, where they encounter high densities of resistant microorganisms (4–6). Their long-distance migratory behaviour enables the secondary introduction of these resistant bacteria into remote or previously unimpacted ecosystems (7, 8). Moreover, the prevalence of resistant bacteria in the vicinity of gull breeding sites has been shown to correlate with human population density and proximity to urban waste sources, suggesting that the extent of AMR within these environments mirrors broader anthropogenic pressures (9).

The transmission of AMR is predominantly mediated by conjugative plasmids, which facilitate horizontal gene transfer across diverse bacterial species. This mechanism is especially prominent in Enterobacterales, where plasmid-mediated genes contribute to the dissemination of clinically relevant resistance traits (10, 11). Plasmids frequently harbour genes encoding the production of extended-spectrum beta-lactamases (ESBLs) and AmpC cephalosporinases, which confer resistance to broad-spectrum beta-lactam antibiotics, as well as genes mediating resistance to quinolones, aminoglycosides, and other clinically relevant antibiotic classes (11–13). Notably, plasmid types carrying such resistance determinants have been identified in bacterial isolates from both wildlife and anthropogenically impacted environments, supporting the hypothesis that antibiotic resistance genes (ARGs) are actively transmitted into natural ecosystems (14–16).

Previous studies (15, 17) on avian hosts have demonstrated that plasmids carrying ARGs can persist within Enterobacterales even in the absence of direct antimicrobial selective pressure. ESBL-producing *Escherichia coli*, in particular, exhibits the ability to survive in the avian gut for extended periods, facilitating environmental shedding and inter-individual transmission among birds as a potential strategy for maintaining colonization (15). Although the exact duration is not established and varies strongly among bacterial species and genotypes, available studies (4, 9) indicate that resistant bacteria can persist in the gut of wild birds for several months. Variation in the persistence of specific *E. coli* sequence types (STs) suggests a disparity in isolate fitness, potentially influenced by the presence of plasmids and their associated fitness costs (17, 18). Plasmids confer evolutionary advantages to STs, such as the ability to produce various bacteriocins, enabling them to outcompete other strains (19).

In this study, we investigated cephalosporin-resistant *E. coli* isolated from the gastrointestinal tract of wild Caspian gull (*Larus cachinnans*) nestlings from a breeding colony at the Nové Mlýny water reservoir in the Czech Republic. Our primary objective was to examine the persistence and colonisation dynamics of resistant strains following the removal of potential environmental sources of resistance. A longitudinal experiment including five gulls housed in controlled captivity, where individuals were sampled periodically over time, was performed. This design enabled us to monitor temporal shifts in *E. coli* ST occurrence and assess their stability. We investigated evolutionary mechanisms potentially underlying the persistence of specific STs through short- and long-read sequencing and phylogenetic analyses. When combined with other employed methods, such as conjugation frequencies, strain fitness, and phenotype analyses, these approaches provided a comprehensive insight into the factors driving strain dynamics. Particular emphasis was placed on the role of colicin, which was encoded by multiple plasmids, including the rapidly disseminating IncI1 plasmid. This plasmid, together with the colicin production, appeared to contribute significantly to the competitive success of certain strains. The study provides novel insights into the dynamics of avian gut colonisation by resistant bacteria, examining persistence over a timescale that has not been previously explored. By extending the observation period beyond earlier research focused on wildlife, it sheds light on how long resistant strains may persist within wild bird populations and highlights the potential role of these hosts as long-term reservoirs.

## MATERIAL AND METHODS

### Sample collection

Cloacal swabs were collected in 2018 from Caspian gulls from a breeding colony inhabiting a small island in the Věstonice region of the Nové Mlýny reservoir (48.895261° N; 16.600583° E), as part of a broader telemetry study (20) conducted during routine bird ringing. This work follows a previous investigation (6) that focused on plasmid-mediated resistance in *Escherichia* in gulls. The sampling site was selected based on several factors: i) the central section of the reservoir is designated as a nature reserve and supports a diverse avian population; ii) the area is directly connected to the remainder of the reservoir, which is primarily used for recreational purposes and therefore subjected to anthropogenic influence; iii) two major rivers flow into the central basin, one of which originates in Brno, the second largest city in the Czech Republic, where municipal wastewater, including hospital effluent, is released into the river system.

Following the initial sampling at the gull colony as part of our previous study (6), five individuals were housed in a wildlife avian facility until they were ready to be released into the wild, equipped with telemetry loggers (20). Initially, each bird was kept in a separate cage; after 1 week, they were transferred to a shared aviary and fed a standardised diet consisting of one-day-old chicken, mice, and small fish. The chicken originated from a hatchery, while the mice and fish were obtained from private breeding facilities. None of the animals used as feed were treated with antibiotics. However, as neither the diet nor the rearing environment was tested for the presence of resistant bacteria, we acknowledge that this may have influenced the results and introduced potential bias into the study. The selected gulls were randomly chosen healthy nonflying juveniles, approximately 1 month old at the time of capture. Nonflying juveniles were selected to ensure minimal prior exposure to anthropogenic environments as the breeding colony was located on an island that limited such contact. Cloacal swabs were collected once at the gulls’ breeding site and subsequently at two-week intervals in the avian facility to monitor longitudinal changes in the resistant bacterial populations. Swabs were placed in AMIES transport medium and cultivated in buffered peptone water. Each bird was sampled on seven occasions, resulting in a total of 35 samples over the course of the study.

### Selective cultivation of resistant *E. coli*

Selective cultivation of peptone water cultures was performed on MacConkey agar (Oxoid Ltd., UK) supplemented with cefotaxime (2 mg/L). From each plate, five to six colonies with morphology consistent with *E. coli* were selected for further analysis. MALDI-TOF mass spectrometry was subsequently used for species identification.

### Beta-lactamase detection

All *E. coli* isolates were tested by polymerase chain reaction (PCR) for the presence of selected beta-lactamase genes, including *bla*_TEM_, *bla*_CMY-2_, and *bla*_CTX-M_. Target genes were chosen based on a previous study (6), in which they were identified as the most prevalent among resistant *E. coli* strains from the same colony. Primer sequences are provided in Table S1.

### Pulsed-field gel electrophoresis (PFGE) and whole-genome sequencing (WGS)

Isolates selected based on PCR results and genomic relatedness, determined by PFGE (21, 22), were subjected to short-read WGS. PFGE macrorestriction patterns were analyzed using BioNumerics v6.6 software (Applied Maths, Belgium) and are presented in Fig. S1. Isolates were grouped into clusters based on 90% similarity, and representative isolates from each cluster were selected for further genomic analysis. Genomic DNA for short-read sequencing was extracted using the NucleoSpin Tissue kit (Macherey-Nagel, Germany). DNA libraries were prepared with the Nextera XT Library Preparation kit (Illumina, USA) and sequenced on the Illumina NovaSeq 6000 platform run in a paired-end mode. Both the DNA library preparation and the sequencing itself were outsourced to the UTS Next Generation Sequencing Facility, Sydney, Australia.

Representative isolates (*n* = 3) were selected for long-read sequencing to determine the location of beta-lactamase and colicin genes and to obtain complete plasmid sequences. Isolate selection was based on short-read sequencing results and focused on STs of greatest interest, with the selection of particular isolate being random. Genomic DNA was extracted using the GenFind V3 kit (Beckman Coulter, USA). Libraries were prepared with the Rapid Barcoding Kit SQK-RBK004 (Oxford Nanopore Technologies, GB), loaded onto a FLO-MIN106 flow cell, and sequenced on the MinION Mk1B platform (Oxford Nanopore Technologies).

### Data analysis and comparative genomics

Raw reads obtained from Illumina sequencing were trimmed using Trimmomatic v0.39 (23) to remove adapter sequences and low-quality read regions (Q ≤ 20). *De novo* assembly was performed with SPAdes v3.13.1 (24) using the “--careful” option. The MLST v2.0.9 (25) tool was utilised for determining the ST of the isolates. ST assignment of nonsequenced isolates was inferred based on PFGE similarity to sequenced representatives. Plasmid replicons, plasmid STs, and ARGs were identified using tools from the Center for Genomic Epidemiology: PlasmidFinder v2.1 (26), pMLST v2.0^26^, and ResFinder v4.0 (27, 28), respectively (https://www.genomicepidemiology.org/services/). Colicin and associated immunity genes were assessed using ABRicate v1.0.1 (https://github.com/tseemann/abricate) with a custom database of genes generated from diverse available databases (VFDB core data set 2025-04-15 (29), VirulenceFinder 2022-12-02 (30), and Bakta 6.0.0 (31).

Raw long reads obtained from Nanopore sequencing were demultiplexed and quality- and adapter-trimmed as described previously (32). The processed reads were *de novo-*assembled using Unicycler v0.5.1 (33) and polished by Racon v1.5.0 (34) and medaka v2.0.0 (35) using the processed long reads and by Pilon v1.2.3 with trimmed short reads. All tools were used with default settings unless stated otherwise. Plasmid sequences of interest were annotated by Bakta v1.10.4 (31) followed by manual curation in Geneious Prime 2024.0.7 (https://www.geneious.com/). The genetic context of colicin and colicin immunity genes carried by plasmids was visualised using clinker v0.0.31(36) and custom R (37) scripts utilising the following packages: tidyverse v2.0.0 (38), ggplot2 v3.5.1 (39), dplyr v1.1.4 (40), and reshape2 v1.4.4 (41).

Plasmids were compared by BRIG v0.95 (42) using short-read assembled genomes mapped to a long-read assembled annotated reference of IncI1 and IncF plasmids. The differences among IncF and IncI1 plasmids from all isolates were assessed using alignment by Snippy v4.6.0 (https://github.com/tseemann/snippy) with corrected short-read data and long-read assembled plasmids as a reference, and the nucleotide similarity was evaluated by snp-dists v0.6.3 (https://github.com/tseemann/snp-dists) The differences among IncI1 were further analysed using Varscan v2.4.6 (43).

Phylogenetic relatedness among isolates was assessed by predicting open reading frames with Prokka v1.14.1 (44), followed by core genome alignment using Roary v3.12.0 (45). This alignment served as the basis for constructing a maximum-likelihood phylogenetic tree in RAxML v8.2.11 (46) under GTR+GAMMA, a model supported by 1,000 bootstraps. Nucleotide similarity between isolates was estimated with snp-dists v0.6.3 (https://github.com/tseemann/snp-dists), based on the number of single-nucleotide polymorphisms (SNPs) in the core genome alignment. The resulting phylogenetic tree was visualised in iTOL v5.7 (47).

### Competition experiments

Overnight cultures of representative isolates of two STs of interest were grown separately in Luria-Bertani (LB) broth (Sigma-Aldrich, USA) at 37°C. The cultures were then diluted to 10^−3^ and incubated at 37°C until reaching an optical density at 600 nm (OD_600_) of 0.6. Equal volumes were subsequently mixed in a 1:1 ratio, while unmixed controls were maintained in parallel. All cultures were incubated for 22 h at 37°C. Following incubation, cultures were serially diluted to 10^−7^ and plated in drops onto LB agar supplemented with cefotaxime. After overnight incubation at 37°C, colony PCR was performed on all resulting colonies to determine the relative abundance of the original strains, as well as their respective transconjugants.

Custom primers targeting chromosomal markers of both STs were designed using Geneious Prime 2023.1.2. The presence of IncF and IncI1 plasmids was evaluated using primers detecting genes *bla*_CMY-2_ and *bla*_CTX-M_. Primer sequences are provided in Table S1.

### Fitness measurements

Isolates of four different STs (*n* = 14) were selected for growth curve analysis (10) to compare their fitness. Selection was based on both the individual host bird and the sampling time point to capture potential diversity and temporal changes in fitness. Isolates were incubated for 24 h at 37°C, with OD_600_ measured every 10 minutes. Growth curves were statistically analysed to estimate the bacterial fitness. OD measurements were recorded using Synergy HT and Synergy HTX plate readers (BioTek Instruments, Inc., USA). Fitness was assessed using area under the curve (AUC) values derived from growth curve data.

### Frequency of plasmid transfer

The same *E. coli* isolates used in the fitness assay (*n* = 14) were selected as donor strains for conjugation experiments. The recipient strain was laboratory *E. coli* MT102 (48), resistant to sodium azide (200 mg/L) and rifampicin (25 mg/L). The plasmid transfer frequency was assessed using the existing protocol for filter mating assays (49). Conjugation was performed in both technical and biological triplicates to quantify the transfer rates.

### Colicin production testing

Colicin production was assessed in the same 14 isolates used for fitness and plasmid transfer frequency experiments. Six indicator strains were selected to represent the most commonly produced colicin types: *E. coli* K12-Row, C6 (ϕ), B1, P400 and 5K, and *Shigella sonnei* 17. The assay followed established protocols (50, 51), with *E. coli* ATCC, A15, and DH5α included as negative controls. A strain was considered a colicin producer if the diameter of the inhibition zone reached or exceeded 1.5 mm.

### Statistical analysis

Statistical computing was carried out in RStudio (52) using custom R(37) scripts and the following packages: readr v2.1.5 (53), magrittr v2.0.3 (54), dplyr v2.4.0 (40), and tidyverse v2.0.0 (38). Data were tested for normality using the Shapiro-Wilk test, followed by the two-tailed Mann-Whitney U test for nonparametric data sets. Significant differences were determined using a *p*-value threshold of α = 0.05. Full statistical results, including test selection and raw data, are provided in Table S2.

## RESULTS

### High variability and dynamics of isolates

A total of 35 samples of cloacal swabs from five birds were collected, yielding 116 cephalosporin-resistant isolates, of which 94 were identified as *E. coli* by MALDI-TOF. From these, 62 isolates were selected for WGS based on PFGE clustering, as well as the bird and time of sampling, in order to cover a broader range of diversity.

**Fig 1:**
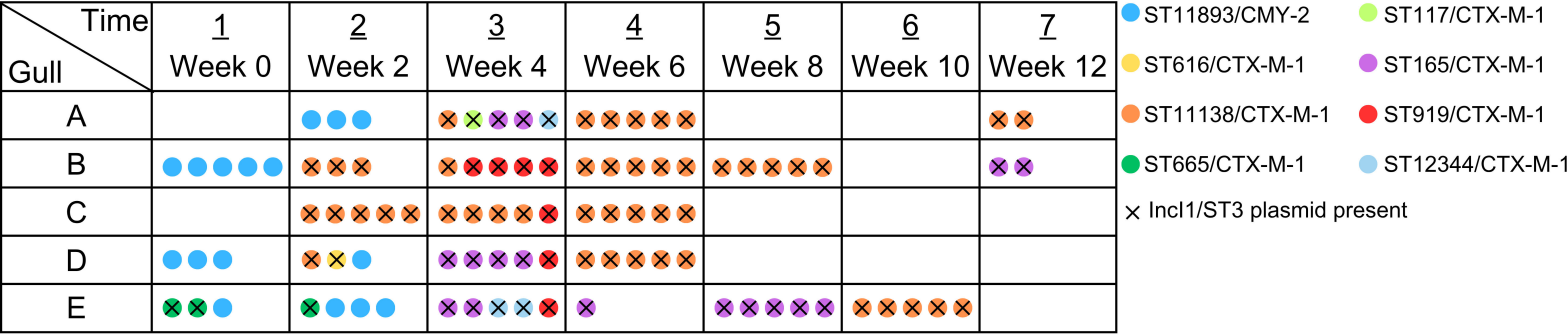
Dynamics and transmission of resistant *E. coli* STs in the gastrointestinal tract of gulls. Each row stands for one gull and each column for one sampling. The colored dots represent *E. coli* colonies of various STs. Colonies carrying the IncI1/ST3 plasmid are marked with an × symbol.

Across the data set, eight different STs were identified (Fig. 1). Shifts in population dynamics as well as transmission of resistance plasmids were observed during the entire experiment. At the beginning (week 0, upon admission to the avian facility), three of the five gulls carried cefotaxime-resistant *E. coli*. The most dominant strain at this time was CMY-2-producing ST11893, accounting for 81.8% (9/11) of the colonies obtained. In addition, only two ESBL-producing ST665 isolates (18.2%; 2/11) were identified in a single bird. These ST665 isolates represented the earliest carriers of the IncI1/ST3/CTX-M-1 plasmid, which subsequently disseminated into other STs.

ST11893 was no longer detected after the second sampling at week 2, having been outcompeted by CTX-M-1-producing isolates carrying the IncI1/ST3 plasmid. The highest ST diversity was observed in week 4, when all birds harboured five STs in total. In the following weeks, ST11138 became dominant in all individuals, representing 50% (47/94) of the *E. coli* isolates collected. Colonisation rates declined significantly by the end of the experiment. In week 10, only one bird carried resistant *E. coli*, and this increased to two birds by week 12. Strain dynamics and patterns suggest transmission between individual gulls, although introduction through feeding cannot be entirely excluded.

Analysis of phylogenetic relatedness revealed no evident branching pattern associated with individual birds or sampling time points. Instead, isolates clustered consistently according to their STs (see Fig. S2). Relatively high diversity was observed even among isolates belonging to the same ST, with the highest variability found in ST11138, which displayed an average difference of 228.46 SNPs, and the lowest in ST11893, with an average difference of 5.04 SNPs. SNP matrices for ST11138, ST11893, ST165, and ST665 are provided in Table S3.

### Phenotypic and molecular traits associated with strain persistence

**Fig 2:**
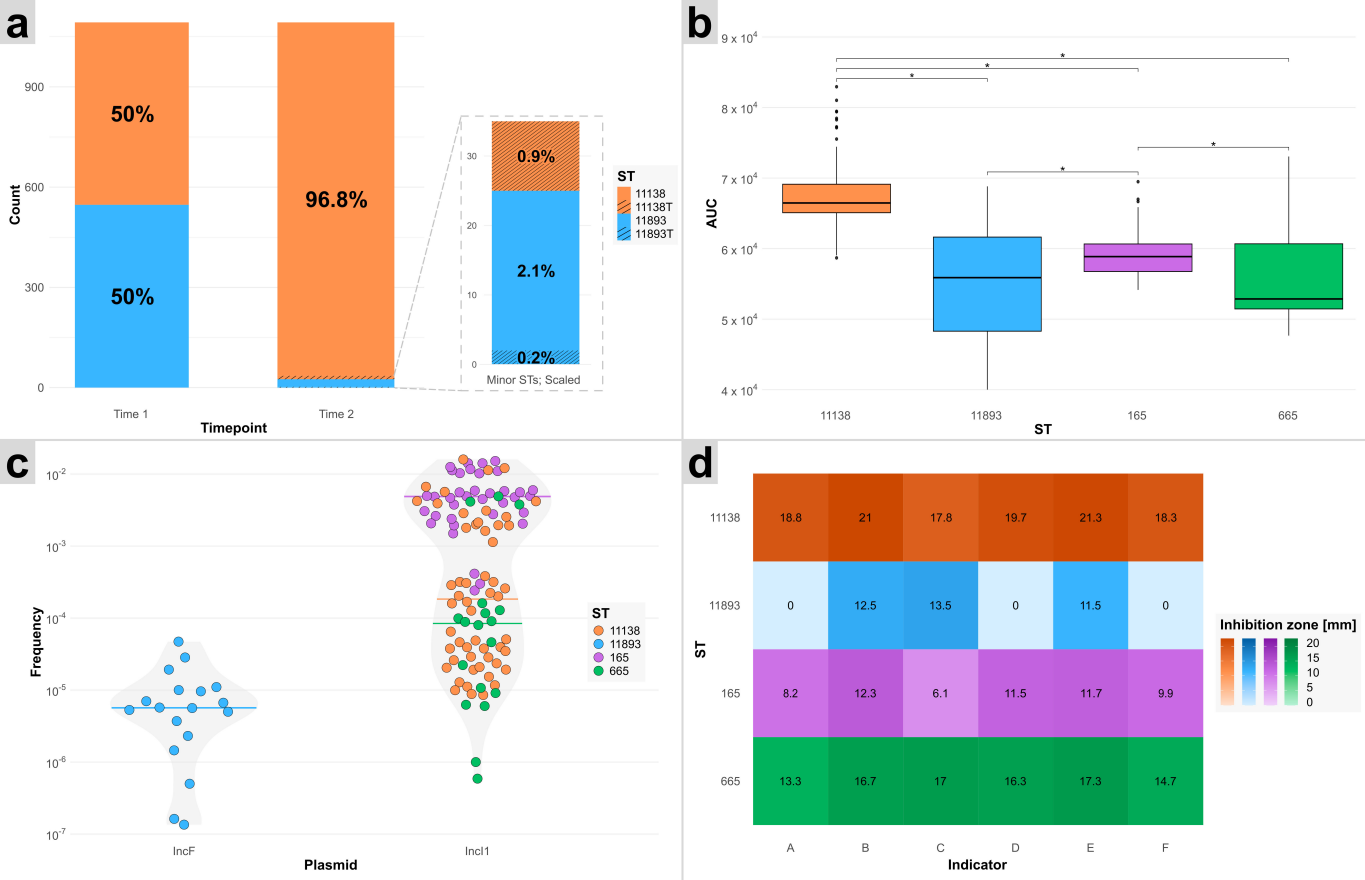
Overview of key comparative assays. (**a**) Results of the competition assays between ST11138 and ST11893, with minor STs enlarged to scale. The “T” in ST11138T and ST11893T describes transconjugant strains. (**b**) Fitness of ST11138, ST11893, ST165, and ST665 based on growth curve analysis. (**c**) Conjugation frequencies of IncF and IncI1 plasmids, grouped by ST, with median values indicated by coloured lines. (**d**) Colicin production profiles, assessed by inhibition of the following indicator strains: A, *E. coli* 5K; B, E. coli B1; C, *E. coli* C6; D, *E. coli* K12-row; E, *E. coli* P400; F, *Shigella sonnei* 17*.*

A single ST, ST11138, repeatedly outcompeted others during the course of the experiment, suggesting a potential selective advantage. To investigate the underlying mechanisms of this dominance, four representative STs were subjected to a set of comparative assays. These included competition experiments, assessments of growth fitness, measurement of plasmid conjugation frequencies, and quantification of colicin production (Fig. 2). These analyses aimed to determine whether the observed advantage was linked to competitive ability, enhanced intrinsic fitness, or increased plasmid transfer frequency. Raw data and *p*-values for all of the analyses are provided in Table S2.

### Competitive dominance of a single ST in competition experiments

The experiment sought to confirm the dominance of ST11138 over ST11893 under controlled laboratory conditions. Competition experiments were conducted to simulate potential interactions between *E. coli* strains within the gull host environment. Two isolates of ST11893 and six isolates of ST11138 were selected for the assay. The primary aim was to assess the dynamics within mixed populations of one ST11893 and one ST11138 isolate, including the potential transfer of the IncF plasmid from ST11893 to ST11138 and of the IncI1 plasmid from ST11138 to ST11893.

Competition assays resulted in a total number of 1,093 colonies analysed by colony PCR. Of these, 96.8% (1,058/1,093) were identified as ST11138 and 2.1% (23/1,093) as ST11893. The rest (1.1%) displayed a conjugative transfer with 0.9% (10/1,093) of ST11138 isolates obtaining the IncF plasmid (ST11138T) and 0.2% (2/1,093) of ST11893 isolates obtaining the IncI1 plasmid (ST11893T) (Fig. 2a). A significant difference (*p*-value < 0.05) was observed between the final counts of most colony types, except between ST11138T and ST11893T, with ST11138 occurring at a notably higher frequency.

### Significant differences in fitness were detected among most STs

Fitness comparisons revealed significant differences between all STs, with the exception of ST11893 and ST665. The highest fitness was observed in ST11138 (AUC 67,346.73) followed by ST165 (AUC 59,167.85), ST665 (AUC 56,602.20), and ST11893 (AUC 55,231.99), which correlated with the *in vivo* observations in live hosts (Fig. 2b).

### Conjugation frequency of the IncI1 plasmid higher than that of the IncF plasmid

A significant difference in conjugation rates was observed between IncI1 and IncF plasmids, with IncI1 being transferred at a 220-fold higher frequency compared to IncF (*p*-value = 0.03318; α = 0.05), explaining a massive IncI1 plasmid spread among all isolates (Fig. 2c). Significant differences in plasmid transfer frequencies were also detected between most STs, with the exception of comparisons involving ST665.

### Dominance of certain STs linked to variability in colicin production

Pairwise comparisons revealed statistically significant differences (*p*-value < 0.05) between multiple STs. ST11893 consistently exhibited low or no colicin production, showing significant differences from other STs across all indicator strains (Fig. 2d). In contrast, ST11138 displayed the highest levels of colicin production, differing significantly from ST165 and ST11893 across all indicators and from ST665 specifically for indicator strains B1 and 5K.

### Varying ecological success linked to plasmid diversity and colicin genes

All sequenced isolates (*n* = 62) carried an IncF plasmid, with the replicon sequence type (RST) specific to the corresponding *E. coli* ST. The most common RST was F24:A-:B1 (53.2%; 33/62), found in all ST11138 and ST616. It was followed by F34:A-:B- (17.7%; 11/62) in ST11893, F-:A-:B73 (11.3%; 7/62) in ST165, F2:A-:B1 (9.7%; 6/62) in ST117 and ST919, F18:A-:B1 (4.8%; 3/62) in ST12344, and F89:A-:B- (3.2%; 2/62) in ST665. A total of 82.3% (51/62) of the isolates, all belonging to STs other than ST11893, also carried an IncI1/ST3 plasmid.

**Fig 3:**
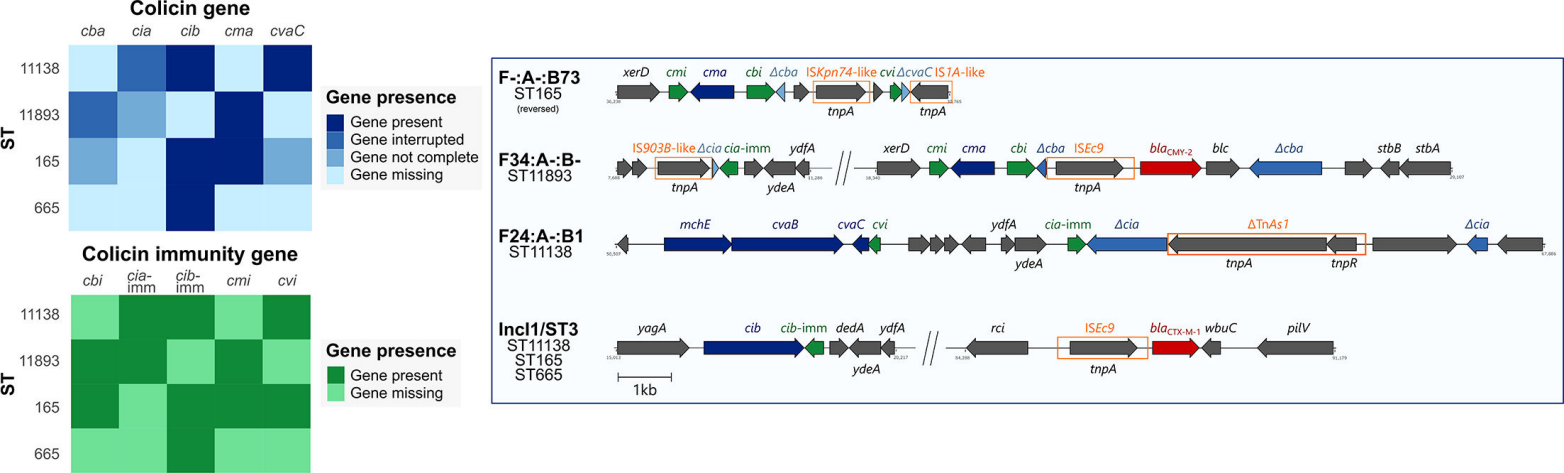
Distribution of colicin and colicin immunity genes across STs and associated plasmids. Heatmaps (left) indicate the presence, disruption, incompleteness, or absence of colicin and immunity genes across different STs. Sequences (right) show the distribution of these genes across plasmids and their corresponding STs. Genes encoding colicin production are highlighted in blue, immunity genes in green, and ARGs in red. Entire insertion sequences are denoted by orange rectangles.

IncF plasmids of various RSTs, as well as the IncI1/ST3 plasmid, carried different combinations of five colicin production genes (*cba, cia, cib, cma,* and *cvaC*) and their respective colicin immunity genes (c*bi, cia*-imm*, cib*-imm*, cmi,* and *cvi*) (Fig. 3). All production genes encode eponymous colicins. The highest number of complete colicin genes was observed in ST11138, which carried *cib* on IncI1/ST3 and *cvaC* on the F24:A-:B1 plasmid, and ST165, harboring *cib* on IncI1/ST3 and *cma* on F-:A-:B73. ST11893 carried *cma* on F34:A-:B-, and ST665 harbored *cib* on IncI1/ST3. Interrupted colicin genes were detected in ST11138 (*cia* on F24:A-:B1, disrupted by a transposase gene) and in ST11893 (*cba* on F34:A-:B-, disrupted by the resistance gene *bla*_CMY-2_). Incomplete genes were found in ST11893 (*cia* on F34:A-:B-) and ST165 (*cba* and *cvaC* on F-:A-:B73). The *cvaB* and *mchE* genes were consistently co-located with the *cvaC* on F24:A-:B1 plasmids as components of a colicin-producing operon. All STs carrying colicin production genes, whether complete, interrupted, or incomplete, also carried the corresponding immunity gene.

**Fig 4:**
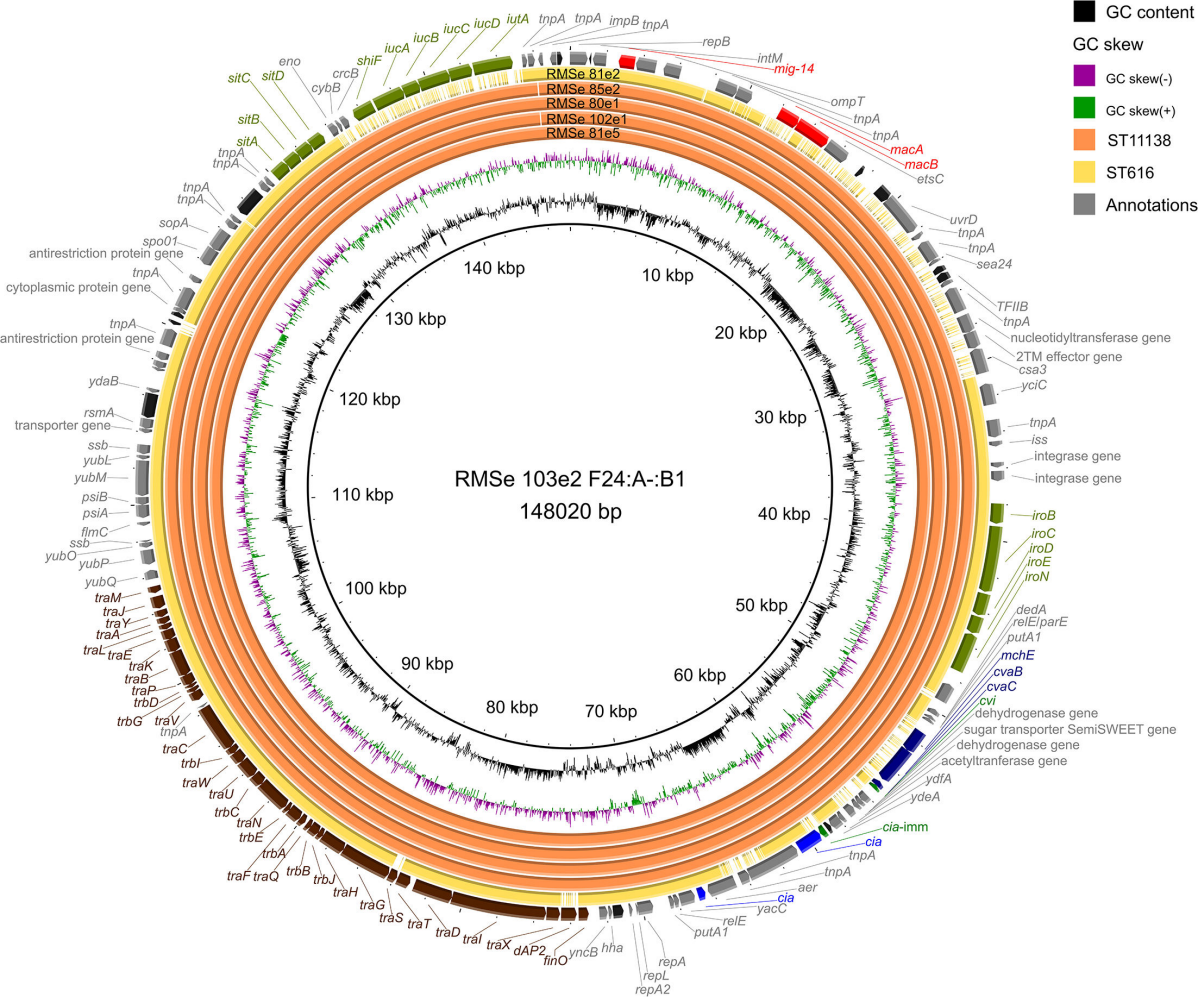
Alignment of the F24:A-:B1 plasmids from selected ST11138 and ST616 isolates. Colicin genes (blue), colicin immunity genes (green), and ARGs (red) are highlighted. Lighter shading indicates disrupted genes. The plasmid transport region is marked in brown and the iron acquisition region in olive green. Hypothetical proteins are represented in black, and other genes are shown in gray. Color assignment of STs is shown in the legend. The F24:A-:B1 plasmid obtained from long-read sequencing of isolate RMSe 103e2 was used as the reference sequence.

The F24:A-:B1 plasmid carried by the ST616 isolate differed distinctly from those found in ST11138 isolates, which exhibited high sequence uniformity (Fig. 4). Notable differences were observed in the presence of several colicin genes (*cia, cia*-imm, and *cvaC*) as well as ARGs (*macA* and *macB*). SNP analysis confirmed the uniformity of the F24:A-:B1 plasmid among ST11138 isolates, with no SNPs detected between them. In comparison, the variant of the same plasmid harboured by ST616 differed by 884 SNPs when aligned against all other sequences (Table S4). For a complete alignment of the F24:A-:B1 plasmids, see Fig. S3.

The F34:A-:B- plasmid, found exclusively in ST11893, exhibited high sequence uniformity across all isolates. In each case, the *cba* colicin gene was disrupted by the *bla*_CMY-2_ resistance gene. A previously collected ST11893 isolate from an earlier study (6) was included for comparison and displayed 100% sequence identity (Fig. S4). Among all analysed plasmids, only one isolate differed by a single SNP; the remaining sequences were identical (Table S4).

The IncI1/ST3 plasmid alignment included representatives from all host STs in this study, as well as ST23 and ST540 sequences from the previous dataset (6). Plasmids from ST117, ST165, ST919, and ST11138 exhibited 100% sequence identity (Fig. S5). In contrast, plasmids from ST616, ST665, ST23, and ST540 showed variation, particularly in transposase-associated regions. SNP analysis of the IncI1/ST3 revealed high overall uniformity, with an average of 2.85 SNPs across all STs (Table S4). The IncI1/ST3 plasmid also carried the complete *bla*_CTX-M-1_ gene for ESBL production in all STs. Due to its carriage of the *cib* colicin gene, absent from all other plasmids, the IncI1/ST3 plasmid was originally thought to be the main driver of the varying strain persistence.

## DISCUSSION

In this study, we provide evidence that Caspian gulls are capable of maintaining cefotaxime-resistant *E. coli* strains in their gut for at least 3 months, continuously shedding ESBL-producing bacteria into the environment and serving as a secondary source for other birds in the flock. This is the longest longitudinal study to date investigating the carriage of antibiotic-resistant bacteria in the gut microbiome of wild migratory birds, offering unprecedented insights into the persistence and dynamics of resistance in a natural host over time. The study also resulted in the longest documented duration of resistant bacteria carriage in wild birds as previous studies have reported gulls shedding colistin-resistant *E. coli* for up to 16 days, after which the bacteria were no longer detected despite continued monitoring (17), and mallards harbouring ESBL-producing *E. coli* for 29 days, with the experiment ending at that point (15). While the number of individuals in our study was lower than in the aforementioned comparable reports involving 15–16 birds, the extended sampling period allowed for a more detailed examination of colonisation dynamics over time. Nevertheless, the small sample size may limit the broader applicability of the findings to wider wild bird populations. Compared to previous studies in mallards (15) and gulls (17), the prolonged colonisation observed in our study may reflect differences in host species, study design, and strain-specific factors. The gull study (17) focused on adult gulls that were artificially inoculated with *mcr-1-*positive *E. coli* and tracked only bacterial shedding, whereas our work tracked juvenile gulls naturally colonised by ESBL-producing strains and assessed both shedding and potential transmission dynamics. Juveniles likely had a more stable gut microbiome, particularly due to colonisation, reduced exposure to external sources, and limited prior antibiotic contact, which may have supported longer bacterial carriage. While the research on mallards (15) observed high transmission rates and stable harbouring of ESBL-producing *E. coli*, the experiment concluded after four weeks, potentially underestimating longer-term persistence.

The initial colonisation patterns suggest that only a subset of individuals carried resistant *E. coli*, with a limited diversity of STs dominated by ST11893. This ST has previously been identified as the most prevalent resistant *E. coli* strain in the same gull colony, detected across multiple individuals during a single-time point sampling (6). Notably, the IncI1/ST3 plasmid was detected in a different ST and in only one gull at this stage, indicating it had not yet become widespread within the group. By week 4, six different STs carrying the IncI1/ST3, likely due to a previously reported ability of the plasmid to disseminate efficiently (55), began to appear, with ST11138 rapidly outcompeting all other strains. The disappearance of ST11893 by the second sampling, along with the absence of other STs lacking the IncI1/ST3 plasmid, may indicate a selective advantage conferred by this plasmid in the captive environment. The observed dominance of ST11138 in our study raises further questions about the contribution of strain-specific versus host-related factors. This ST has not been detected in the previous study of gulls or their environment at the same site (6), suggesting it was not part of the established local microbiome. Its successful persistence and competitive advantage may reflect a combination of functional traits, such as efficient colicin production, and compatibility with the gull gut environment under the specific conditions of captivity. Whether this strain would show similar dominance in other bird species or in free-living conditions remains unexplored. Further comparative studies across hosts and settings would help clarify the ecological scope of ST11138 and its potential as an emerging avian-associated lineage.

Although overall colonisation rates declined by the end of the experiment, shedding was still observed along with seemingly ongoing transmission of strains between individual gulls, indicating a continued risk of environmental dissemination and spread to other hosts. Unlike *in vitro* experiments or *in silico* models, our study tracked the persistence and transmission of resistant bacteria in live wild birds under biologically relevant conditions, providing ecologically grounded evidence of strain dynamics and plasmid spread. While the lack of testing of the diet represents a limitation of this study, we are confident that transmission between gulls also contributed to the observed dynamics. All gulls received the same diet, yet not all were colonised by the same strains, and carriage declined near the end of the experiment, with some birds remaining completely free of resistant bacteria despite continued dietary exposure. These findings suggest that the observed colonisation patterns cannot be fully explained by potential external introduction alone.

Due to the observed dominance of a single ST, four STs (ST11893, ST11138, ST165, and ST665) were selected for further analyses to investigate the mechanisms behind this phenomenon. Our study was the first to report the existence of ST11138, with only two other genomes currently available in EnteroBase (enterobase.warwick.ac.uk), both isolated in 2020 from poultry in the USA. This limited distribution supports its novelty and raises the possibility of recent introduction or niche adaptation. The outcome of the competition assay suggests a marked competitive advantage of ST11138 over ST11893 under the tested conditions, consistent with its observed dominance in the host population. Although plasmid transfer of IncF and IncI1 was also detected, conjugation rates were low and unlikely to have influenced the outcome. The high fitness of ST11138, demonstrated both in direct competition and individual growth measurements, likely contributed to its long-term persistence in the host population. In contrast, the limited growth capacity of ST665 and ST11893 may explain their inability to establish or maintain colonisation beyond the early stages of the study. While we did not perform plasmid curing experiments, our findings align with prior reports suggesting minimal fitness costs associated with IncI1/ST3 plasmids (55, 56) and are supported by the successful persistence of strains harbouring this plasmid regardless of their overall fitness levels. This suggests that factors other than IncI1/ST3 plasmid carriage were responsible for the observed differences in strain success.

None of the tested STs harboured IncF plasmids with identical RST, further highlighting that plasmid type alone cannot fully explain the observed differences in persistence. Notably, the IncF plasmid exhibited markedly lower transfer efficiency than the IncI1/ST3 plasmid, which may have limited its dissemination potential. Among the tested STs, ST165 appeared particularly effective in promoting the spread of the IncI1/ST3 plasmid, followed by ST11138, suggesting that the host background may influence conjugation dynamics. Notably, these two STs were the only ones consistently detected in the gulls from week 6 onward. Genomic analyses suggested that colicin production could contribute to ST persistence, prompting us to perform assays focused on evaluating bacteriocin activity. Colicins and other bacteriocins have previously been shown to provide a competitive advantage to their producers as opposed to strains that lack the genes for their productions (57–59). As expected, ST11893 displayed no inhibitory activity against three indicator strains, suggesting a lack of production for three of the six colicins tested, as opposed to the other STs, which showed broader activity. This absence of bacteriocin production is likely a key factor contributing to the disappearance of ST11893. In contrast, ST11138 exhibited the highest colicin production, supporting its prevalence over the other STs. To our knowledge, colicin-mediated persistence has not been previously investigated in the context of specific STs, and no studies to date have explored how differences in bacteriocin production among STs may influence the long-term colonisation in natural host populations.

The presence of colicin and colicin immunity genes was further investigated using long-read analysis, which allowed precise localisation of these genes on individual plasmids. ST11893 was the only ST lacking the *cib* gene, which was located on the IncI1/ST3 plasmid in all other STs. This absence corresponds with observed reduced colicin activity and likely contributed to the early disappearance of ST11893, which was outcompeted by various emergent STs with the IncI1/ST3 plasmid. Notably, the *cib* region was conserved across all isolates harbouring this plasmid regardless of the ST, suggesting strong plasmid stability and horizontal dissemination. Although ST11893 was the only ST carrying a complete *cba* gene, which could have potentially served as a competitive mechanism, it was located on an F34:A-:B- plasmid and disrupted by the *bla*_CMY-2_ gene and therefore did not provide the strain with any evolutionary advantage. The same plasmid, including the disruption, was found in ST11893 isolates collected from multiple birds during a previous study (6) at the same gull colony. The gulls analysed in our study were, however, physically separated from the colony throughout the study, indicating that the strain with the exact plasmid managed to persist in the bacteria even with no outside contact with the colony, which could otherwise serve as a secondary source. Although the F34:A-:B- plasmid uniquely harbored the *cma* gene region, its associated colicin activity appeared insufficient to support persistence of ST11893 in the presence of more competitive STs. In contrast, the F24:A-:B1 plasmid, consistently found in ST11138, likely played a key role in its dominance, potentially due to the presence of the *cvaC* gene, which was absent or incomplete in other strains. The genetic uniformity of this plasmid among ST11138 isolates, alongside its divergence in the less successful ST616, further supports its role in enhancing strain fitness. Meanwhile, the F-:A-:B73 plasmid carried by ST165, which also persisted throughout the experiment, shared similarities with the F34:A-:B- plasmid in its *cma* region but may have conferred a greater selective advantage through its co-occurrence with the IncI1/ST3 plasmid carrying the *cib* gene region, which was absent in ST11893.

Together, these findings demonstrate that the persistence and competitive success of resistant *E. coli* strains in wild gulls are shaped by a complex interplay of plasmid-associated traits and ecological dynamics. While plasmid carriage alone did not correlate with strain fitness, the presence of specific colicin and colicin immunity genes emerged as a key factor influencing long-term colonisation. These patterns underscore the importance of bacteriocin-mediated interactions as a selective force within microbial communities and support the view that mobile genetic elements contribute not only to AMR dissemination but also to competition among strains within wildlife reservoirs. Moreover, colicin production associated with AMR is particularly found in epidemic plasmid lineages found in multidrug-resistant bacteria. These conclusions are consistent with recent evidence showing that colonisation history and host ecological context strongly influence the evolution of AMR in the gut microbiome (60). Our results support the idea that early colonising strains equipped with strong competitive traits, such as colicin production, are more likely to persist and dominate, highlighting the importance of microbial community dynamics in shaping resistance patterns even in the absence of selective antibiotic pressure.

While our research provides important insights into the long-term carriage and dynamics of cephalosporin-resistant *E. coli* in wild birds, several limitations should be acknowledged. The focus on a single resistance phenotype limits broader conclusions regarding other clinically relevant resistance types. Additionally, the study was conducted on a small number of individuals from a single breeding site, without incorporating the influence of migratory behaviour or environmental variation across habitats. These factors may affect the general applicability of our findings. Future research would benefit from expanding the study design to include multiple AMR phenotypes, diverse ecological settings, and sampling across different stages of the birds’ migratory cycle to provide a more comprehensive view of AMR persistence in wildlife.

In summary, our study shows that the long-term persistence of cephalosporin-resistant *E. coli* in Caspian gulls is shaped not simply by plasmid carriage or initial prevalence but by a multifaceted combination of bacterial fitness, plasmid dissemination efficiency, and competitive traits such as colicin production. These features enabled certain strains, notably ST11138 and ST165, to dominate the gut microbiome and sustain colonisation over time, even without ongoing antibiotic selective pressure. By capturing these processes in live wild birds under naturalistic conditions, albeit with potential anthropogenic influence introduced through standardised feeding, and across an extended period, our study provides rare and ecologically grounded insights into how AMR is maintained outside clinical or laboratory settings. This unique host-based perspective shows that resistance dynamics in wildlife reservoirs are governed as much by microbial interactions and evolutionary persistence strategies as by the presence of ARGs themselves. Importantly, our findings underscore the ecological novelty of longitudinal *in vivo* tracking of AMR persistence in natural hosts and highlight the need to consider ecological and microbiome-level interactions when assessing the environmental dimension of AMR under the One Health concept. Beyond its ecological insights, our study also holds relevance for AMR prevention and control strategies. The long-term shedding of resistant strains by wild birds highlights the potential role of breeding colonies as reservoirs and environmental sources of resistance. These findings support the need for regular microbiological monitoring, particularly in areas with proximity to human activity or agricultural lands. Such surveillance could serve as an early warning tool to detect high-risk resistance phenotypes and guide targeted interventions, such as managing food waste exposure or reducing environmental contamination around migratory birds’ habitats. Incorporating wild bird populations into broader One Health AMR surveillance frameworks may be critical to understanding and mitigating resistance flow across ecosystems.

## Data Availability

All whole-genome sequencing (WGS) data have been deposited under BioProject number PRJNA1144792. The long-read sequences are associated with BioSample accession numbers SAMN54157616, SAMN54157617, and SAMN54157618.

## SUPPLEMENTAL MATERIAL

Figure S1 msystems.01663-25-s0001.png Photo of PFGE gel.

Figure S2 msystems.01663-25-s0002.pdf Phylogenetic tree of sequenced isolates.

Figure S3 msystems.01663-25-s0003.docx BRIG of all F24:A:B1 plasmids.

Figure S4 msystems.01663-25-s0004.docx BRIG of all F34:A-:B- plasmids.

Figure S5 msystems.01663-25-s0005.docx BRIG of all IncI1/ST3 plasmids.

Supplement legends msystems.01663-25-s0006.docx Legends to supplemental material.

Table S1 msystems.01663-25-s0007.xlsx Sequences of used primers.

Table S2 msystems.01663-25-s0008.xlsx Statistical methods and results overview.

Table S3 msystems.01663-25-s0009.xlsx SNP matrix of different sequence types.

Table S4 msystems.01663-25-s0010.xlsx SNP matrix of different plasmids.

ASM does not own the copyrights to Supplemental Material that may be linked to, or accessed through, an article. The authors have granted ASM a non-exclusive, world-wide license to publish the Supplemental Material files. Please contact the corresponding author directly for reuse.
