# Supplementary figures and images for: From ecology to evolution: plasmid- and colicin-mediated persistence of antibiotic-resistant Escherichia coli in gulls
Source: mSystems. 2025 Dec 29;11(2):e01663-25. doi: 10.1128/msystems.01663-25 (PMC12911396; doi:10.1128/msystems.01663-25)

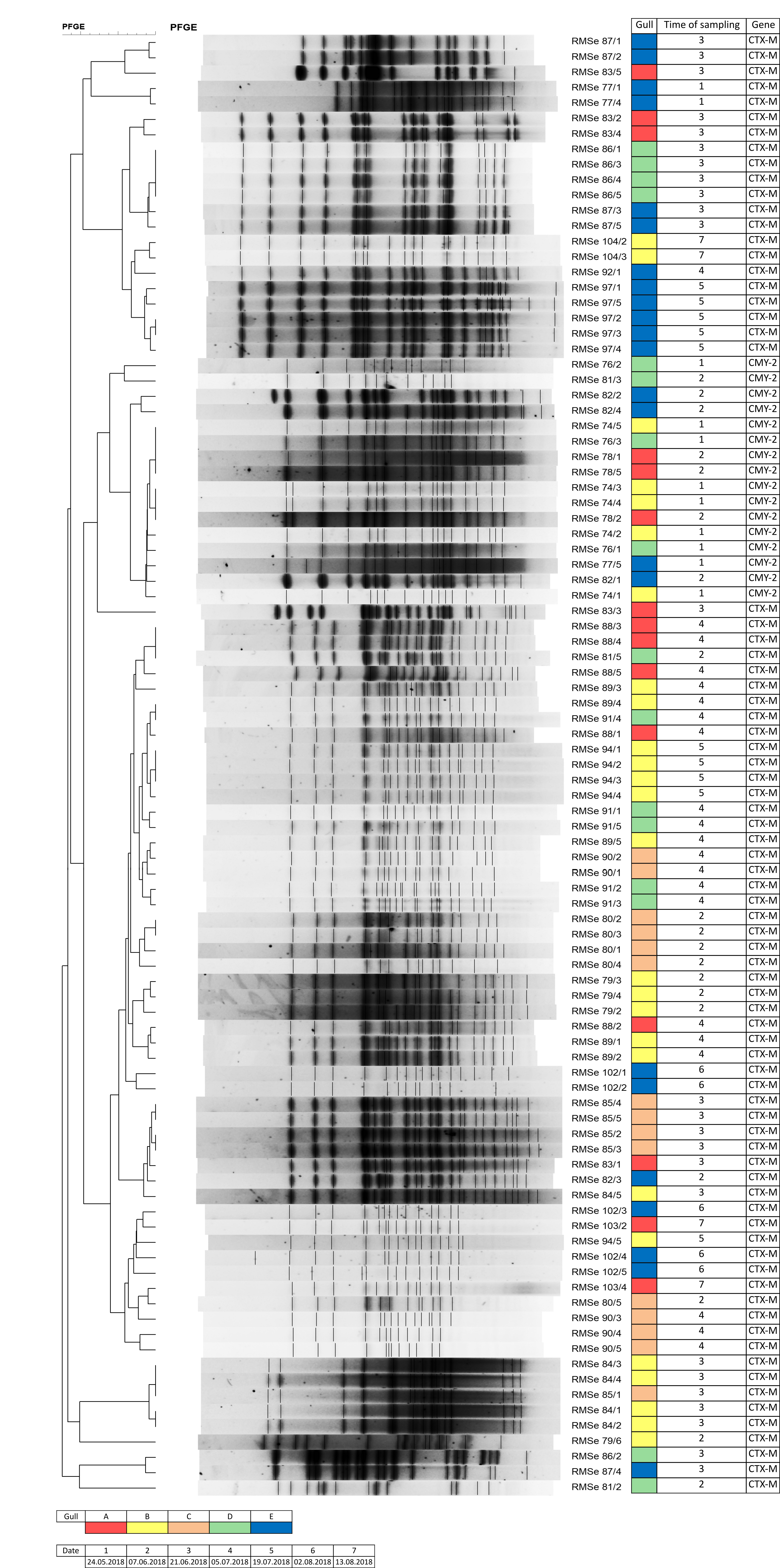

Supplement: Figure S1 — Photo of PFGE gel. [file msystems.01663-25-s0001.png]

Tree scale: 0.01

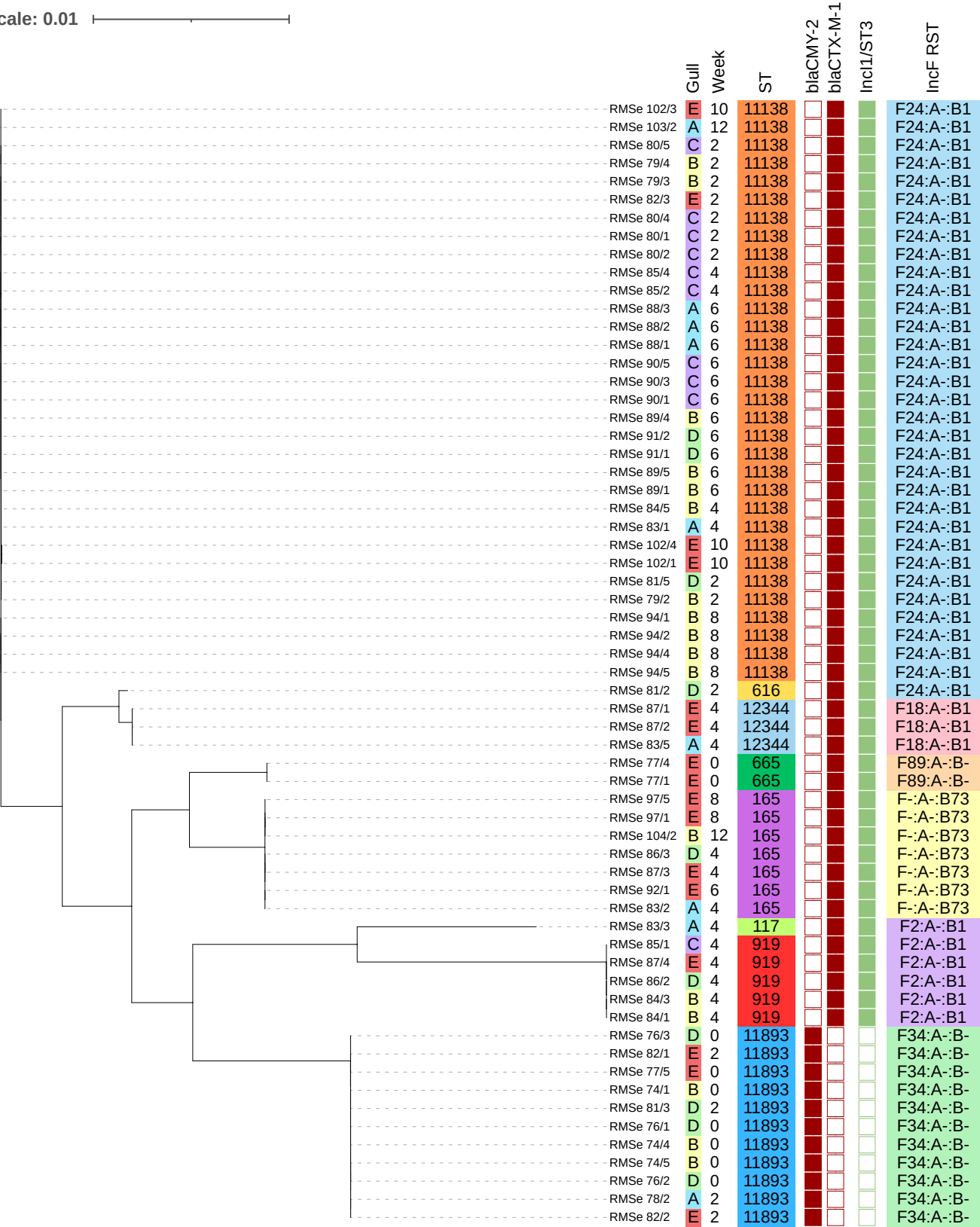

Supplement: Figure S2 — Phylogenetic tree of sequenced isolates. [file msystems.01663-25-s0002.pdf]

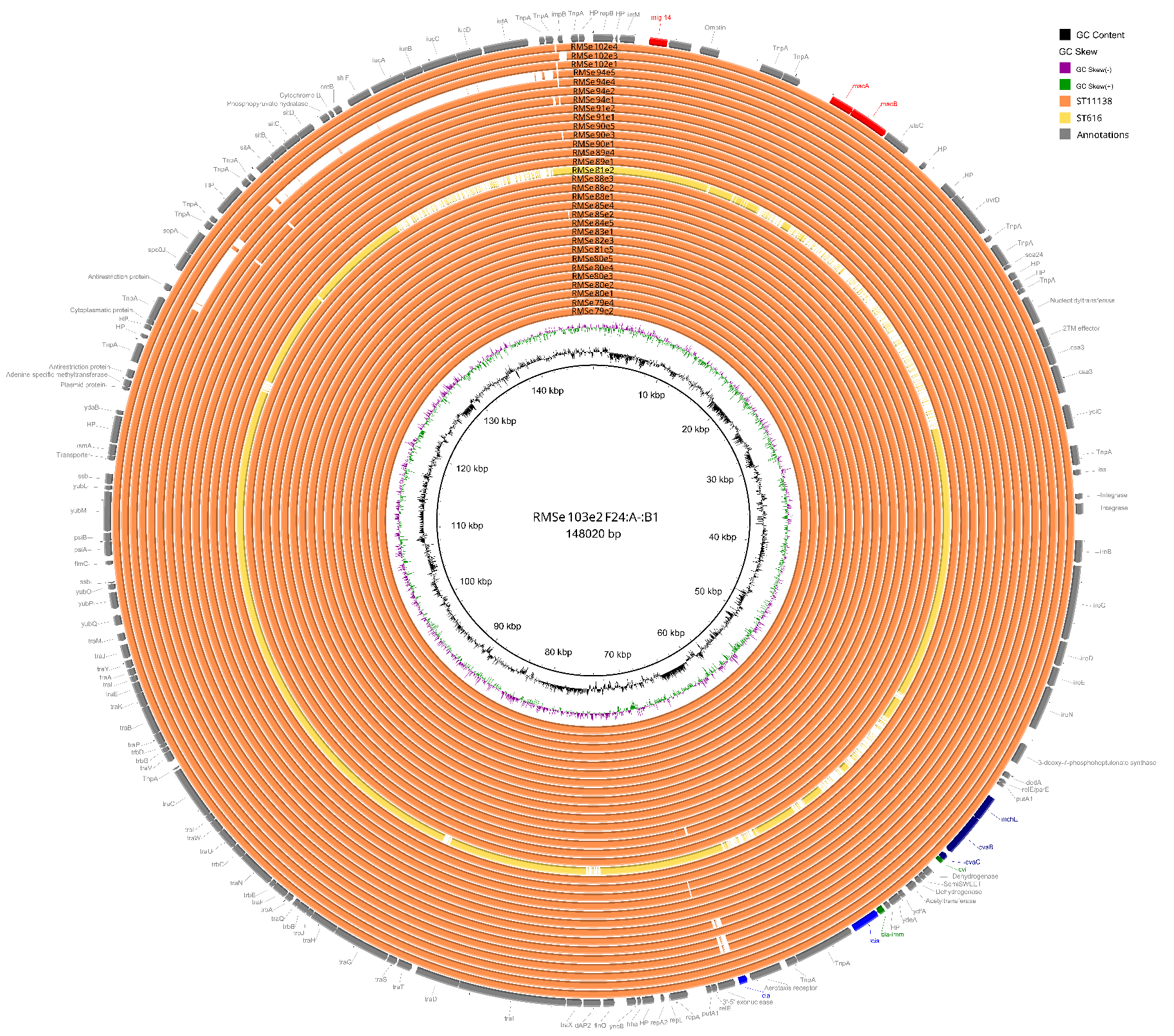

Supplement: Figure S3 — BRIG of all F24:A:B1 plasmids. [file msystems.01663-25-s0003.docx]

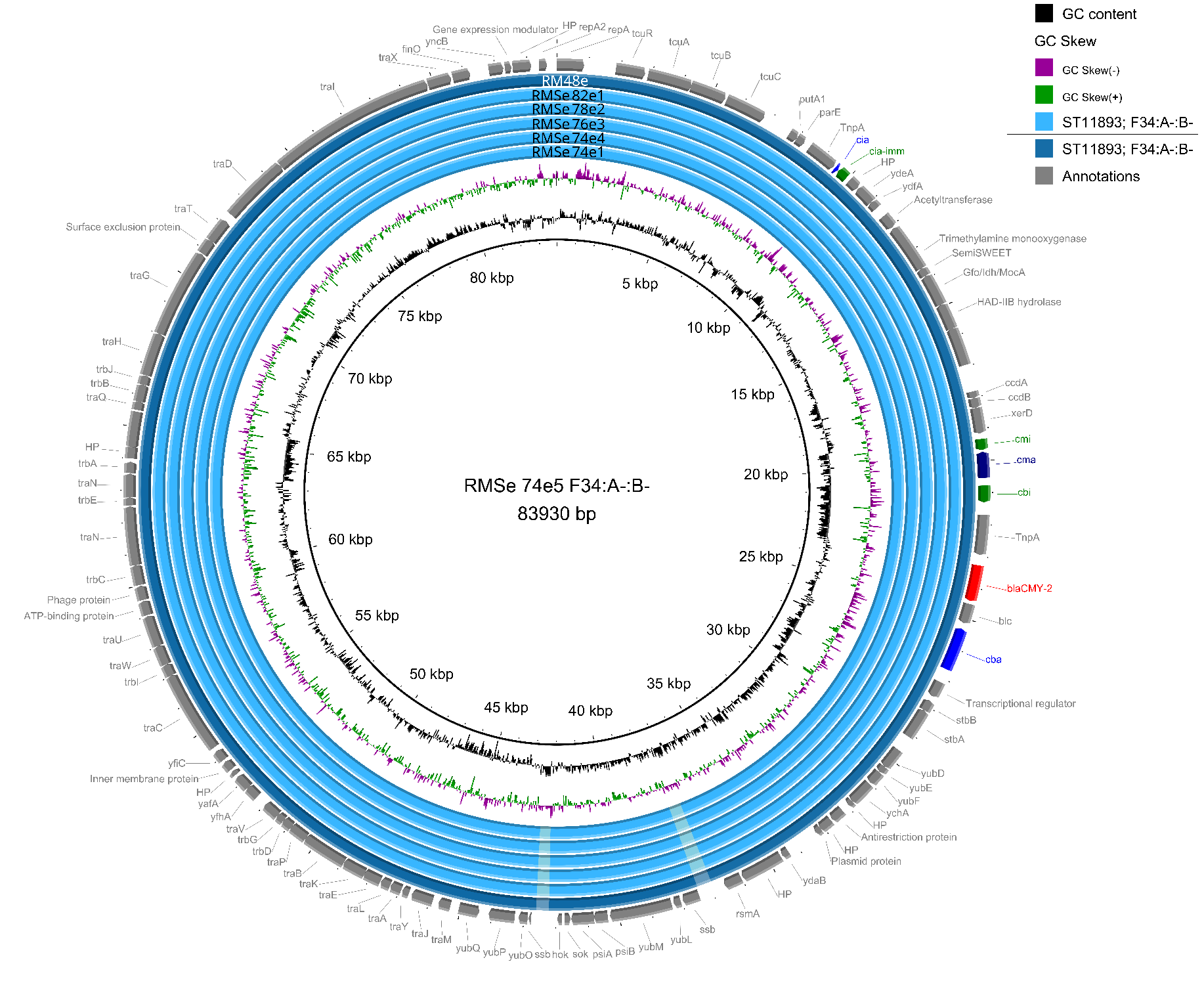

Supplement: Figure S4 — BRIG of all F34:A-:B- plasmids. [file msystems.01663-25-s0004.docx]

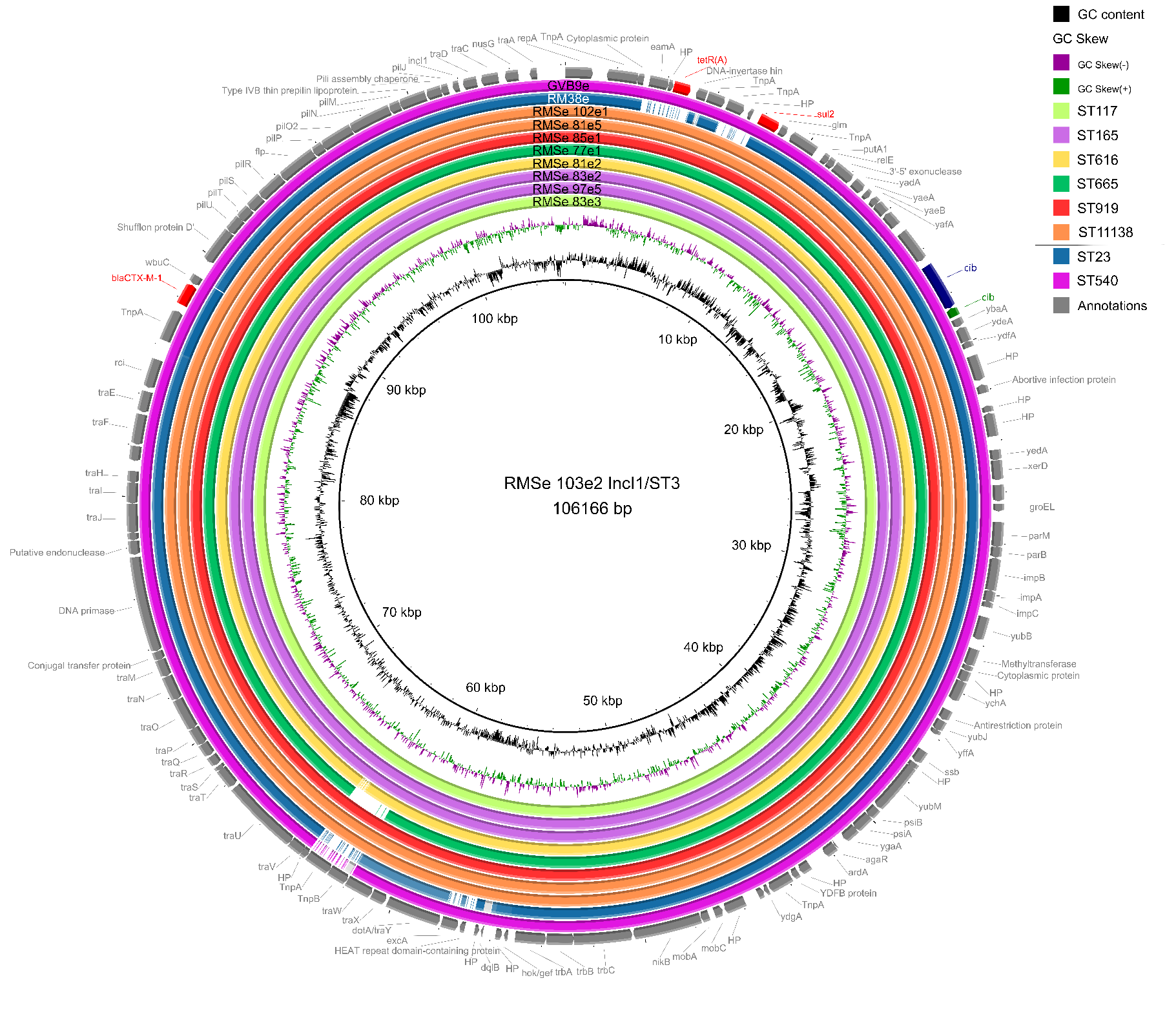

Supplement: Figure S5 — BRIG of all IncI1/ST3 plasmids. [file msystems.01663-25-s0005.docx]
