# Supplementary material for: From ecology to evolution: plasmid- and colicin-mediated persistence of antibiotic-resistant Escherichia coli in gulls
Source: mSystems. 2025 Dec 29;11(2):e01663-25. doi: 10.1128/msystems.01663-25 (PMC12911396; doi:10.1128/msystems.01663-25)
Supplement: Supplement legends — Legends to supplemental material. [file msystems.01663-25-s0006.docx]

Table S1 – Sequences of used primers

Table S2 – Statistical methods and results

Table S3 – SNP matrix of different sequence types

Table S4 – SNP matrix of different plasmids

Figure S1 – Photo of PFGE gel

Figure S2 – Phylogenetic tree of sequenced isolates

Figure S3 – BRIG of all F24:A-:B1 plasmids

Figure S4 – BRIG of all F34:A-:B- plasmids

Figure S5 – BRIG of all IncI1/ST3 plasmids
